# Rare variants in Fanconi anemia genes are enriched in acute myeloid leukemia

**DOI:** 10.1038/s41408-018-0090-7

**Published:** 2018-06-01

**Authors:** Kyaw Ze Ya Maung, Paul J. Leo, Mahmoud Bassal, Debora A. Casolari, James X Gray, Sarah C. Bray, Stephen Pederson, Deepak Singhal, Saumya E. Samaraweera, Tran Nguyen, Gökhan Cildir, Mhairi Marshall, Adam Ewing, Emma L. Duncan, Matthew A. Brown, Russell Saal, Vinay Tergaonkar, Luen Bik To, Paula Marlton, Devinder Gill, Ian Lewis, Andrew J Deans, Anna L Brown, Richard J D’Andrea, Thomas J Gonda

**Affiliations:** 1 Basil Hetzel Institute for Translational Health Research, The Queen Elizabeth Hospital, Woodville, SA Australia; 2 School of Medicine, University of Adelaide, Adelaide, SA Australia; 3 Department of Haematology and Oncology, The Queen Elizabeth Hospital, Woodville, SA Australia; 4 Centre for Cancer Biology, University of South Australia and SA Pathology, Adelaide, SA Australia; 5 Institute of Health and Biomedical Innovation, Translational Research Institute, Queensland University of Technology and University of Queensland Diamantina Institute, Brisbane, QLD Australia; 6 School of Pharmacy and Medical Sciences, University of South Australia, Adelaide, SA Australia; 7 Departments of Haematology, SA Pathology and Royal Adelaide Hospital, Adelaide, SA Australia; 8 Adelaide Bioinformatics, School of Biological Sciences and School of Mathematics, University Of Adelaide, Adelaide, SA Australia; 9 Mater Research Institute, University of Queensland, Brisbane, QLD Australia; 10 Department of Endocrinology, Royal Brisbane and Women’s Hospital, Brisbane, QLD Australia; 11 School of Medicine, University of Queensland, Brisbane, QLD Australia; 12 Institute of Molecular and Cell Biology, NUS School of Medicine, Singapore, Singapore; 13 Clinical Haematology, Princess Alexandra Hospital Brisbane, Woolloongabba, QLD Australia; 14 St Vincent’s Institute of Medical Research, Melbourne, VIC Australia; 15 Department of Genetics and Molecular Pathology, SA Pathology, Adelaide, SA Australia; 16 University of South Australia Cancer Research Institute, Adelaide, SA Australia; 17 School of Pharmacy, University of Queensland, Brisbane, QLD Australia

Acute Myeloid Leukemia (AML) is an aggressive hematological malignancy caused by somatically acquired changes affecting a well-defined set of genes^1^. While rare high-risk variants affecting specific transcription factors account for a proportion of myelodysplastic syndrome (MDS) and AML associated with a family history, the contribution of other germline variants conferring low-intermediate risk has not yet been determined, partly because these are more difficult to identify from pedigree analysis. Here we use an Australian AML patient cohort to analyze rare, deleterious variants affecting genes involved in the rare recessive bone marrow failure syndrome Fanconi Anemia (FA). FA is caused by bi-allelic germline mutations in any of the 22 FANC genes (except for *FANCB* and *FANCR* which are X-linked and autosomal dominant), and is associated with profoundly increased risk of AML^2^. The proteins encoded by the FANC genes participate in the removal of interstrand crosslinks (ICL) and the protection and resolution of stalled replication forks, an essential step for faithful DNA replication^2,3^. Deficiency for these genes, combined with other mutations, results in pre-leukemia or leukemia in mouse models^4^.

Novel, rare, somatic and germline, coding and splicing FANC gene variants (Tier 1 mutations^1^, MAF < 0.001) were identified using whole exome sequencing (WES) of 131 samples from adult Caucasian AML patients (cohort characteristics shown in Supplementary Table S1). Variants were also identified in WES data from an ethnically matched, all-female, healthy control cohort (*n* = 323)^5^. Following pathogenicity filtering, we identified a total of 53 heterozygous FANC variants in 45 patients (Supplementary Table S2). In contrast to what has been observed for FA AML, for which over-representation of +1q, del7/7q and +3q has been reported^6^, we observed significant under-representation of monosomy 7/del(7q) (*P* = 0.028; Fig. 1a and Supplementary Table S1).

**Fig. 1:**
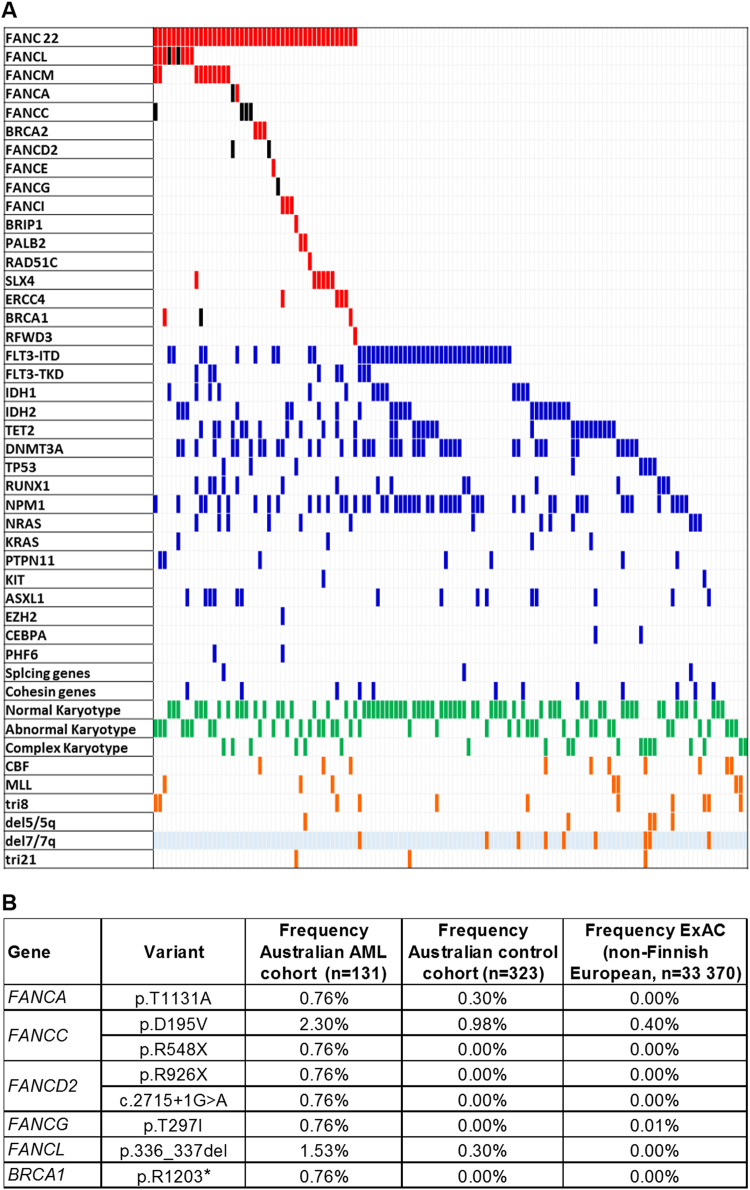
FANC variants in the Australian AML cohort. **a** Association of FANC mutations with recurrent somatic AML mutations and cytogenetics in the Australian AML Cohort. The columns in the figure represent each patient in the Australian AML cohort (*n* = 131). The first row in the figure represents the 22 FANC genes as a group. The subsequent rows represent individual FANC genes, AML recurrently mutated genes, karyotype and cytogenetic characteristics. Patients carrying one or more FANC mutations (*n* = 44) are represented with red boxes. Black boxes indicate patients carrying a D-C mutation for the specific FANC gene. Patients with specific AML mutations are represented with blue boxes. Patients with normal, abnormal (1 or 2 abnormalities) and complex (3 or more abnormalities) karyotypes are represented with green boxes. Patients with specific karyotypic abnormalities are represented by orange boxes. Del7/7q is under-represented (blue highlight) in the FANC-mutant AML patient group (*P* = 0.028). **b** D-C mutations present in the Australian AML cohort. Frequency of each D-C mutation is presented for the Australian AML and control cohorts, and for the ExAc (non-Finish European) database

Sanger sequencing of selected FANC tumor variants, for which matched non-tumor material was available, showed that the majority were present in the non-tumor material (11 out of 12, Supplementary Fig. S1), indicating that AML FANC variants are predominantly germline. Further sequencing of cDNA from selected AML samples revealed expression of both wild type (WT) and mutant sequences for the majority of variants (Supplementary Fig. S2). A number of FANC variants of significant interest were also identified based on known disease associations, protein structure, or clinical characteristics (summarized in Supplementary Table S3**)**.

For a stringent analysis of pathogenic FANC variants, we next identified known disease-causing (D-C) variants in the AML and healthy cohorts by cross-referencing to pathogenic variants in the FA (FAMutdb: http://www2.rockefeller.edu/fanconi/) and breast cancer (kConFab: http://www.kconfab.org/Index.shtml and BIC: https://research.nhgri.nih.gov/bic/) databases (Fig. 1b). For case-control comparison with our healthy all-female cohort, we limited analysis to female AML patients. While the overall frequencies of female patients and controls with FANC variants were similar (35% vs. 32%, respectively), we observed an elevated frequency of female patients carrying D-C variants [13.7% vs. 4.5% controls; Odds Ratio (OR) = 3.3, 95% CI: 1.3–8.6; *P* = 0.018]. We also observed increased frequency of D-C FANC variants in the Australian and TCGA AML cohorts compared to that reported for healthy male and female European-Americans in the ESP database (*n* = 4298 individuals)^7^ (Australian cohort: 6.9% vs. 2.1% controls, OR = 3.4, 95% CI: 1.7–7.0, *P* = 0.0003; TCGA cohort: 4.9% vs. 2.1% controls, *P* = 0.054). The frequency of damaging variants for several FANC genes (*FANCC*, *FANCL,* and *FANCM*, Supplementary Fig. S3) was significantly increased in the Australian AML cohort relative to that in the non-Finnish European population in the Exome Aggregation Consortium (ExAC) database (Supplementary Table S4). Finally, comparison of all rare damaging variants in both the Australian and TCGA AML cohorts to the healthy Australian control cohort, using burden analysis, showed an elevated frequency of *FANCL* variants in the Australian AML cohort (*P* = 0.01, Supplementary Fig. S4), and of *FANCC* and *FANCO* variants in the TCGA cohort (*P* = 0.037 and *P* = 0.01, respectively), consistent with a previous report^8^. Taken together, the elevated frequencies of D-C and damaging FANC variants suggest that subtle changes to FA pathway function in HSC may play a role in AML predisposition and pathogenesis. There are a number of important differences that need to be considered when relating our study to previous studies of FA families which have not found increased incidence of AML in FA carriers^9,10^. Firstly, such familial studies are biased towards analysis of only 3 FANC genes (*FANCA*, *FANCC*, and *FANCG*), which represent the vast majority of FA cases. It is possible that bi-allelic loss of function for some FANC genes may not be tolerated at the germline level (i.e., those genes rarely found mutated in FA) while rare heterozygous mutations may still confer a cancer risk for carriers. Secondly, while familial studies address the question of high-risk D-C FANC variants in carriers, modest-risk or low-risk variants in these genes would be predicted to induce AML with low penetrance, masking the familial pre-disposition. Thus, further analysis focusing on assessing familial cancer and AML incidence for families across all FA complementation groups, and additional prospective sequencing studies in larger multi-center AML and control cohorts, will help to clarify this issue. Such studies are also important to establish the implications for FA families, particularly for selection of sibling donors for stem cell transplantation of FA patients, and for identification of carriers who may have modest-intermediate risk of AML or other cancers. Other studies have reported damaging germline FANC variants in MDS/sAML, therapy related AML, and in cohorts of patients with a range of other familial cancers (Supplementary Table S5).

To investigate whether heterozygous FANC mutations can indeed impair the cellular response to DNA damaging events, we generated MCF10A clones with heterozygous *FANCL* deficiency using CRISPR-Cas9. We compared responses to treatment with an ICL agent (mitomycin C, MMC) for a single WT clone, 3 independent heterozygous *FANCL*-mutant clones, and a bi-allelic *FANCL*-mutant clone carrying a nonsense mutation on one allele and a single amino acid deletion on the other (Supplementary Fig. S5). FANCL protein level showed a 75% reduction compared to the WT control for the bi-allelic *FANCL* clone, and 35–45% reduction for the heterozygous clones (Supplementary Fig. S6). Treatment with MMC did not alter the cell cycle distribution of the heterozygous clones compared to the WT clone; however, the bi-allelic clone displayed accumulation of cells in G2/M phase (Supplementary Fig. S7), consistent with delayed repair of ICLs. Moreover, while the WT clone displayed robust FANCD2-foci formation following MMC treatment, this was not observed for the bi-allelic *FANCL*-mutant clone (Fig. 2a–c), consistent with loss of FANCL activity^11^. Importantly, for the three heterozygous clones, the frequencies of cells lacking FANCD2 foci (Fig. 2b) and with ≥ 3 FANCD2 foci (Fig. 2c) were intermediate compared to the WT and bi-allelic clones indicating reduced FANCL activity in the heterozygous clones compared to WT, but more activity compared to the bi-allelic mutant clone. Overall, these data are consistent with a reduction in, but not abrogation of, DNA repair activity in cells carrying heterozygous damaging mutations in *FANCL*. While in this study we focused on FA DNA repair pathway activation in response to ICL-induced DNA damage, recent reports indicate a role for some FANC genes in replication fork protection^3^; therefore, it will also be of interest to measure the response of FANC heterozygous cells under conditions of replicative stress. It is well established that *BRCA1/2* haploinsufficiency is associated with an impaired DNA damage response, telomere erosion, genomic instability, and premature senescence^12^, and further detailed functional studies of other FANC heterozygous models are now important to link the epidemiological and functional data.

**Fig. 2:**
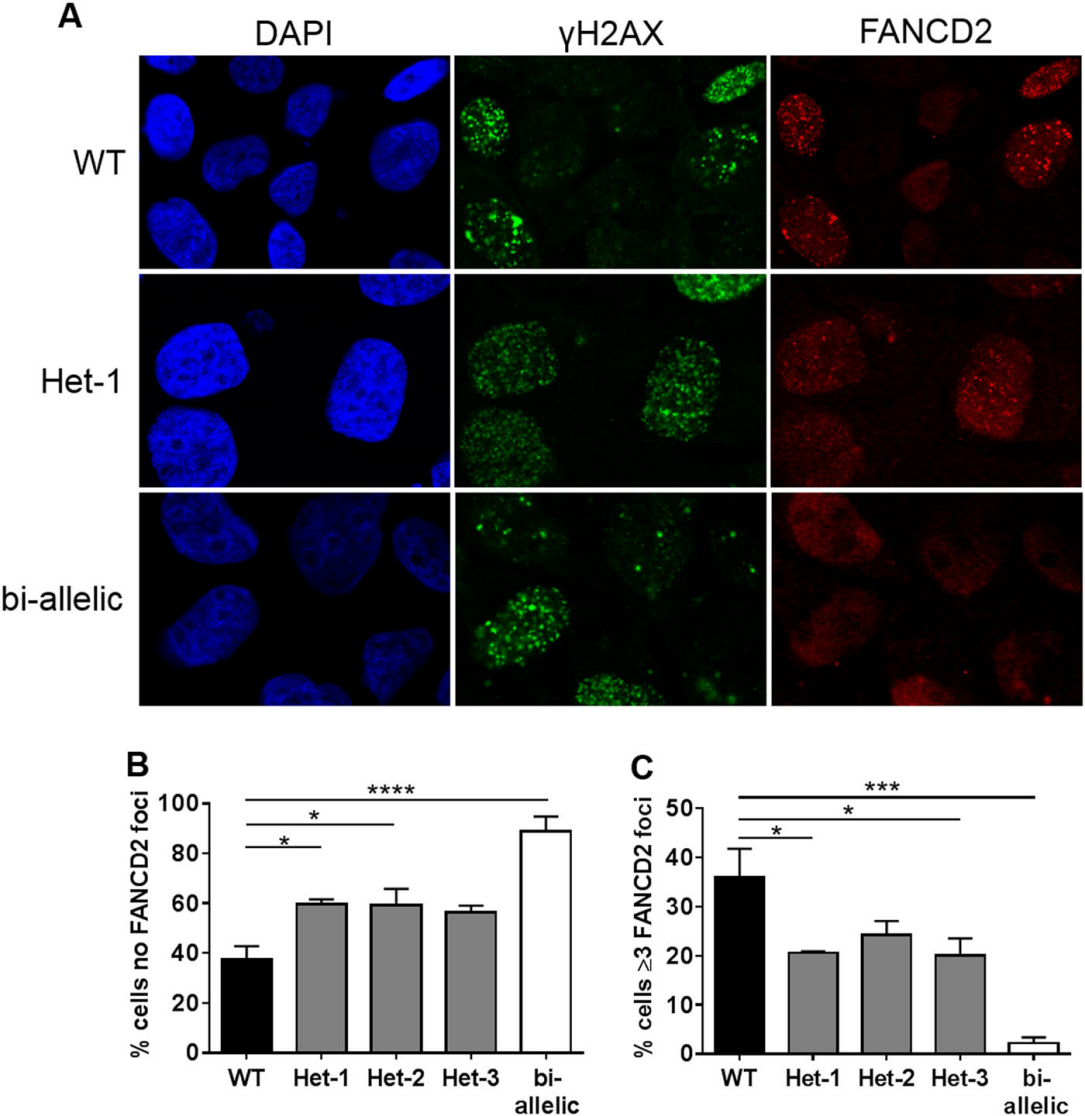
FANCD2 foci formation in a *FANCL* heterozygous cell line model. **a** Immunofluorescent images captured at 63 × magnification for three representative MCF10A CRISPR-*FANCL* clones; WT, heterozygous (Het-1), and bi-allelic. Cells were treated with mitomycin C (MMC) and probed with DAPI (blue) or antibodies for γH2AX (green), and FANCD2 (red). **b** Percentage of γH2AX positive cells with no detectable FANCD2 foci in MCF10A CRISPR-*FANCL* clones. Black: WT clone, gray: heterozygous clones (Het-1, Het-2, Het-3); white: bi-allelic clone. For statistical comparison, One-way ANOVA with Tukey multiple comparison was performed. *P < 0.05, ***P < 0.001, ****P < 0.0001. **c** Percentage of γH2AX positive cells with FANCD2 foci ≥ 3 in the 5 MCF10A CRISPR-*FANCL* clones. Black: WT clone, gray: heterozygous clones (Het-1, Het-2, Het-3); white: bi-allelic clone. For statistical comparison, One-way ANOVA with Tukey multiple comparison was performed. *P < 0.05, *** *P* < 0.001, **** *P* < 0.0001

In summary, our findings suggest that decreased function of the FA DNA repair pathway in HSC is due to deleterious germline heterozygous FANC variants which may result in a reduced capacity to maintain genome integrity, which may in turn contribute to increased risk of AML. This may be particularly significant under conditions associated with increased replicative stress or DNA damage. Such a deficiency would be predicted to have important consequences during emergency hematopoiesis^13^ or when HSC are confronted with endogenous or exogenous cross-linking toxins^14^. The effect of gene variants affecting the FA pathway will also be influenced by other factors, such as environmental exposure, infectious agents and/or, other genetic variants that affect metabolism of aldehydes, or individual DNA damage response. Over time, a subtle change to genomic stability in HSC may contribute to acquisition of somatic driver mutations explaining enrichment of pathogenic FA variants in AML cases. Finally, our findings may have therapeutic implications. Recent studies have demonstrated that the FA pathway, together with Poly-ADP ribose polymerase 1 (PARP1), play a key role protecting replication forks and preventing genomic instability during replication^3^. Thus, AML cells with partial FA pathway deficiency may display enhanced sensitivity to agents that boost replicative stress and DNA damage, particularly when combined with PARP1 inhibitor; this combination has been proposed as a potential therapeutic approach in AML^15^.

## Electronic supplementary material

Supplementary Information

Supplementary table S2
