# Supplementary material for: Rare variants in Fanconi anemia genes are enriched in acute myeloid leukemia
Source: Blood Cancer J. 2018 Jun 1;8(6):50. doi: 10.1038/s41408-018-0090-7 (PMC6002376; doi:10.1038/s41408-018-0090-7)
Supplement: Supplementary file 1 — Supplementary Information [file 41408_2018_90_MOESM1_ESM.docx]

**SUPPLEMENTARY INFORMATION**

**Rare variants in Fanconi Anemia genes are enriched in Acute Myeloid Leukemia**

**Maung *et al***

**TABLE OF CONTENTS:**

1. **Supplementary Materials and Methods**
2. **Supplementary Figures**
   - **Supplementary Figure S1**
   - **Supplementary Figure S2**
   - **Supplementary Figure S3**
   - **Supplementary Figure S4**
   - **Supplementary Figure S5**
   - **Supplementary Figure S6**
   - **Supplementary Figure S7**
   - **Supplementary Figure S8**
3. **Supplementary Tables**
   - **Supplementary Table S1**
   - **Supplementary Table S2**
   - **Supplementary Table S3**
   - **Supplementary Table S4**
   - **Supplementary Table S5**
4. **Supplementary References**
5. **SUPPLEMENTARY MATERIALS AND METHODS**

**AML samples**. AML samples were obtained from the Australian Leukaemia & Lymphoma Group (ALLG) tissue bank at the Princess Alexandra Hospital (PAH, Brisbane, QLD, Australia) and from the SA Cancer Research Biobank (SACRB) at the Royal Adelaide Hospital (RAH) and SA Pathology (Adelaide, SA, Australia). The use of the samples for this research study was approved by the PAH, the RAH, the University of Adelaide, the University of South Australia and the University of Queensland Human Research Ethics Committees (HRECs: HREC/05/QRCH/77, HREC/04/QPAH/172 and HREC/13/RAH/612). ALLG AML specimens were collected with informed consent. AML patient samples obtained from 1998 onwards from SACRB were collected with signed informed consent for research purposes. The RAH HREC waived the requirement for informed consent for SACRB specimens collected before 1998.

**Culture of bone marrow mononuclear cells (BMMNC), mesenchymal stromal cells (MSC) of patient samples and genomic DNA extraction**. Patient bone marrow samples (BMMNC) were thawed in a 37^o^C water bath and resuspended in 20mL of resuspension media consisting of Iscove’s Modified Dulbecco’s Medium (IMDM; Sigma) with 20% fetal bovine serum (FBS; JRH Biosciences) supplemented with an additional 2mM L-glutamine (Sigma), 100U/mL penicillin and 100μg/mL streptomycin (Sigma) and 50U/mL DNAse I (Sigma). The resuspension media was added dropwise at a steady rate. The samples were spun for 5 minutes, supernatant was discarded and the cell pellets were resuspended in the recovery media consisting of IMDM (Sigma) with 10% FBS (JRH Biosciences), supplemented with 2 mM L-glutamine (Sigma), 100U/mL penicillin and 100μg/mL streptomycin (Sigma), 10ng/mL hIL-3 (Peprotech: 200-03), 10ng/mL hTPO (Peprotech: 300-18), 50ng/mL hSCF (Peprotech: 300-07), 10ng/mL hFLT3-L (Peprotech: 300-19) and 20ng/mL hIL-6 (Peprotech: 200-06). Viability of each sample was determined using 0.4% Trypan Blue (Sigma) and haemocytometer. The samples were recovered incubated in a Sanyo humidified incubator overnight with 5% CO_2_ at 37^o^C. After recovery, suspension cells consisting mainly of blast cells were lysed and genomic DNA was extracted using the QIAmp DNA Blood Mini Kit (QIAGEN) as per manufacturer’s recommended protocol.

Mesenchymal stromal cells (MSC) which had adhered to the tissue culture flask after recovery of the patient bone marrow samples, were cultured in Alpha-Modification Minimum Essential Medium Eagle (A-MEM; Sigma) with 20% FBS (JRH Biosciences) supplemented with 100μM L-ascorbic acid (Wako), 2mM L-glutamine (Sigma), 100U/mL penicillin and 100μg/mL streptomycin (Sigma). Once sufficient numbers of MSC were obtained, cells were detached using 1xTrypsin (Sigma), followed by extraction of genomic DNA using the QIAmp DNA Blood Mini Kit (QIAGEN) as per manufacturer’s recommended protocol.

**Control Cohort.** For case-control studies comparing mutation frequencies to that in healthy individuals we used WES data generated from a normal healthy cohort (n=799) (ref 1). This control cohort is all female of Caucasian ethnic origin with mean age of 67yrs (46-86yrs). These samples have been sequenced on the Illumina TrueSeq Enrichment kit v2.0 (Illumina, San Diego, CA, USA) and analysed using the same methods as described below. For the Australian AML patients, 323 of these controls with a mean coverage of (27.5x) were used for the burden analysis against the AML cohort. In addition, a cohort of 49 germline samples, sequenced using Illumina Nextera Rapid exome capture, were used to control for potential bias between the two capture kits, but were not used in the burden analysis (see methods below). For the replication study with TCGA AML sequence data, we used the other 476 samples from the healthy cohort, these had a mean coverage of 23.9x.

**Whole Exome Sequencing of AML samples**. Genomic DNA was extracted from patient AML samples or mesenchymal stromal cells using the QIAamp DNA Blood Mini Kit (Qiagen, Hilden, Germany) according to the manufacturer’s instructions. Genomic DNA was sonicated and DNA sequencing libraries were constructed using a preparation kit for paired-end sequencing (Illumina) as per the manufacturer’s protocol. Liquid-phase hybridization for exome capture was performed using the Illumina TruSeq Exome Enrichment Kit v2.0 (Illumina) (n=89) or Illumina Nextera Rapid (FC-140-1003, Illumina) (n=35). Efficiency of sequence capture was assessed using quantitative real-time PCR with standard control primers as recommended by the manufacturer and quality of each DNA sequencing library was assessed using a 2100 Bioanalyzer and DNA 1000 chip kits (Agilent Technologies, Santa Clara, CA, USA). DNA concentration was standardized to 10 nM for sequencing. Massive parallel sequencing was performed using the Illumina HiSeq 2000 configured for paired-end reads. The 89 Illumina TrueSeq AML exomes had a mean coverage of 57x (26-102x), while the 35 Illumina Nextera AML exomes had a mean coverage of 47.6x (22-144x). Exome sequencing and variant calling was performed at the UQ Centre for Clinical Genomics.

**Base calling and variant filtering**. Initial base calling was performed using the CASAVA 1.7 data analysis pipeline software (Illumina). Sequence data were aligned to the current build hg19 of the human genome using the Novoalign alignment tool [V2.07.09 1] (ref 2). Sequence alignment files were converted using SAMtools [v0.1.14] (ref 3) and Picard tools (v1.42). SNPs and indels for all 131 AML samples were simultaneously called using the best practice protocols described for Genome Analysis Toolkit (GATK v3.5-2 for Australian AML samples and GATK v3.2-2 for TCGA replication samples). This genotyping included sample level Indel realignment and variant quality score recalibration (VSQ). Genotypes were annotated using ANNOVAR^4^ using Refseq, Ensembl and UCSC transcript definitions and the Ensembl Variant Effect Predictor (VEP). Sequencing data were analyzed and filtered using custom scripts employing R and Bioconductor. Good quality SNPs and indels (VSQ: FILTER=PASS) were retained. We describe additional sample and genotype level filtering applied for the burden analyses below.

For several of the subsequent analyses we defined rare variants as those with a population frequency/minor allele frequency (MAF) <0.001 reported in dbSNP147, 1000 genomes (April 2012 version, any ethnicity) and the 6500 NHLBI-ESP project (any ethnicity). We include monomorphic variants and those reported in dbSNP without a population frequency in this category. Deleterious variants were defined if ANNOVAR (using any of the 3 transcript definitions), or the VEP, predicted a mutation to be non-synonymous, splicing (including splice donor and splice acceptor variants defined by VEP), stop-gain, stop-loss, a frameshift or non-frameshift substitution or deletion, an initiator codon variant, a stop retained variant or an incomplete terminal codon variant. We additionally excluded loci where coverage was low and/or the missing rate was high across the cohort. These loci were identified by first filtering genotypes with less than 7 reads supporting heterozygous calls, and 2 for homozygous calls, before calculating the missing rate. If the missing rate exceeded 80%, all genotypes at that loci were excluded. This approach removes low coverage regions where genotypes can be unreliable and discrimination between homozygous and heterozygous calls is poor. We are aware that sequencing artifacts can occur even with this filtering, often appearing as novel or rare mutations (in a population sense) occurring at high frequency in a genotyped cohort. To help identify these, we applied a Hardy-Weinberg p-value filter of 10^-6^ to the controls and additionally excluded loci where the allele frequency in the control genotypes greatly exceeded that of the population frequency filter. We defined this as 6 standard deviations away from the population threshold of 0.001. Under the binominal approximation, this is defined by (“control allele count”- n*p)/sqrt(n*p*(1-p))<=6. For example, if n=400 and *P*=0.001, this restricts our analysis to loci with 3 or less alleles reported in the control cohort. We additionally excluded loci where the mean genotype quality (GQ) score was <50 in samples where an alternative allele was predicted. To control for potential biases generated by different exome capture methods and different cohorts, we compared both genotype counts between control groups and modelled the background allele frequency between the different cohorts. If the genotype counts differed between Illumina and Nextera control cohorts under the binomial approximation with *P*<0.001, we excluded that loci. We modeled the background allele frequency for all loci using samples with homozygous reference genotypes, essentially using the alternative allele frequency to estimate a position dependent background sequencing error rate within each cohort. Differences in that background error rate were used to identify loci where potential batch effects might occur. We chose to exclude loci where the background error rates differed by 6 standard deviation in loci where there was a least 100 reads from the cohorts. Control cohorts on Illumina and Nextera Captures were compared in the analysis for the Australian AML cases and background error rates between control and TCGA was made in the replication experiment. One loci in the FANC gene group failed this filtering in the analysis for the Australian AML cohort, but none in the TCGA cohort analysis. For further pathogenicity filtering we used the Combined Annotation Dependent Depletion algorithm (CADD score >10) (ref 5). The frequencies of known recurrent somatic AML mutations identified in our AML cohort showed excellent concordance with that from the TCGA^6^ (r=0.886, **Supplementary** **Figure S8**), providing validation of our sequencing and mutation calling methods, and demonstrating high sensitivity for variant detection. Selected FANC gene variants were validated in matched diagnostic and germline material by conventional Sanger sequencing to establish somatic status.

**Analysis of FANC variant enrichment in AML cases vs controls**. To compare the frequency of uncommon, deleterious, protein coding variants affecting the FANC gene group (22 genes) and individual FANC genes, we performed burden analysis between the Australian AML patient cohort (n=131) and the ethnically matched (Caucasian) healthy female control population (n=323). Burden analysis^7^ was performed using the SkatMeta program in R with no allele weighting. Because the control cohort was all female, we excluded genes on the X or Y chromosomes to remove any gender bias. Ethnicity was confirmed by converting the control and AML samples to PLINK genotype format and merging with 1000 genome control samples of known ethnicities. After removing regions with known long-range LD, principle component analysis using shellfish (http://www.stats.ox.ac.uk/~davison/software/shellfish/shellfish.php) was then preformed to remove ethnic outliers (greater than 6 standard deviation from the weighted mean of Caucasian 1000 genome samples). Residual population stratification was controlled for by using the first 4 principle component eigenvectors as covariates in the burden tests. However, we found that results did change significantly if Caucasian samples where used in burden test without additional population correction using the eigenvectors, suggesting that the remaining 131 AML cases and 323 controls were ethnically matched.

For comparison of the TCGA AML cohort with healthy individuals, we compared variants identified in the published sequences from an ethnically-matched (reported as Caucasian) cohort of 102 AML cases from the TCGA consortium.^6^ Genotyping was performed using the haplotype callers (version 3.0), again with GATK best practices and otherwise annotated and analyzed using the same pipeline and quality control procedures as above.

**Identification of Disease-causing (D-C) mutations.** D-C mutations were identified by cross-referencing to the FA database (Rockefeller University, FAMutdb; http://www.rockefeller.edu/fanconi/), the Kathleen Cuningham Foundation Consortium for research into Familial Breast (kConFab; http://www.kconfab.org/Index.shtml), and the National Institute of Health (NIH)’s Breast Cancer Information Core (BIC) database (https://research.nhgri.nih.gov/bic/). Mutations annotated as FA haplotype or highlighted in red in the FA database (breast cancer associated), annotated as pathogenic in the kConFab database, or annotated as Class 5 mutation in the BIC database were classified as D-C. As the healthy Australian control cohort was all-female, and there have been reports of over-representation of variants for some FANC genes in females^8^ we limited comparisons of D-C variants to female AML patients only.

**Odds ratio calculation.** Odds ratios were calculated using GraphPad Prism 6 software (GraphPad Software, La Jolla, CA, USA).

**Cell culture.** MCF10A cells were cultured using Dulbecco's Modified Eagle's Medium nutrient mixture F-12 HAM (DMEM-F12; Sigma), with 5% horse serum (Sigma) supplemented with 20ng/mL epidermal growth factor (EGF; R&D Systems), 500ng/mL hydrocortisone (Sigma), 10μg/mL insulin (Sigma), 100ng/mL Cholera toxin (Sigma), 100U/mL penicillin and 100μg/mL streptomycin (Sigma).

**MCF10A CRISPR model of *FANCL* heterozygosity.** Three different single-guide RNA (sgRNA) were designed using the http://crispr.mit.edu/ online tool and cloned individually into the pSpCas9(BB)-2A-Puro (PX459) V2 construct that contained Cas9. The CRISPR-Cas9 construct was transiently transfected into the MCF10A cell line, and transfected cells were selected for 2 days using antibiotic selection (puromycin). Single cells were isolated using limiting dilution and expanded to generate clonal lines. A total of 50 viable clones were obtained and gDNA extracted from each. T7 endonuclease assay and Sanger sequencing were used to determine the presence of deletions at the *FANCL* locus. TOPO TA (Thermo Fisher Scientific) cloning was performed following manufacturer’s protocol and DNA from colonies of transformed DH5α cells was sequenced to confirm *FANCL* mutation and purity of each selected MCF10A CRISPR clone.

**Cell cycle analysis.** 2x10^5^ cells were seeded in each well of a 6-well plate and left to recover overnight. 40ng/mL of MMC or vehicle (DMSO) were added to the wells and incubated for 48 hours. The cells were harvested and resuspended in 300µL of cold 1x PBS. Cells were fixed with 700µL of 100% ethanol added to each tube dropwise (with vortexing). The tubes were incubated at 4^o^C for 30 minutes, spun down and resuspended in 100µL of propidium iodide solution (4µg/mL). The tubes were incubated at 37^o^C for 30 minutes (in the dark). Fluorescence was measured in a Beckman Coulter Gallios, using Gallios Cytometry List Mode Data Acquisition and Analysis Software version 1.2 (Beckman Coulter). Results were analyzed with FCS Express 4 Flow Research Edition software (De Novo Software) using the Multicycle setting.

**Western blot.** Cells were harvested and lysed with NP40 Lysis buffer (ThermoFisher Scientific) supplemented with cOmplete™ protease inhibitor (Roche), PhosSTOP™ (Roche) and Pefabloc^®^ (Roche) at the recommended concentrations. Protein was quantified using the DC Protein Assay kit (BioRad) as per manufacturer’s protocol. 100µg of lysate were loaded for SDS-PAGE. Protein was transferred from the gels into PVDF membranes using a semi dry transfer apparatus (BioRad). The membranes were blocked for 1h at room temperature with 5% skim milk in 0.1% Tris-buffered saline + 0.1% TritonX-100 (TBS-t) and then incubated with primary antibodies overnight at 4^o^C. Antibodies were diluted as follows: anti-FANCL(B-11) (Santa Cruz Biotechnology, Dallas, TX, USA) 1:200 in 5% skim milk in 0.1% TBS-t, and anti-α-tubulin(E-19)-R (Santa Cruz Biotechnology) 1:2000 in 5% BSA in 0.1% TBS-t. After incubation with secondary antibodies for 1h at room temperature membranes were scanned in a Typhoon FLA 9000 (GE Healthcare) scanner.

**Immunofluorescence assay.** In each well of an 8-well chambered slide, 7500 cells were seeded and left to recover for 72 hours. The cells were treated with 40ng/mL of MMC or DMSO (vehicle) for 48 hours. The wells were washed using 1x PBS. The cells were fixed using 4% paraformaldehyde for 20 minutes and washed using 1x PBS. The cells were permeabilized using 0.3% Triton™ X-100 solution for 10 minutes and washed using 1x PBS. The wells were blocked using the blocking solution (10% FBS and 0.1% NP40 in 1x PBS) for 1 hour. Primary antibodies for FANCD2 rabbit polyclonal antibody (Novus: NB100-182) and γ-H2AX mouse monoclonal antibody (MERCK-Millipore: 05-636-I) were diluted 1:1000 in blocking solution, added to the wells and incubated for 3 hours on a rocking shelf at room temperature. The secondary antibodies goat α-mouse Alexa488 (Cell Signalling: 4408) and goat α-rabbit Alexa647 (Cell Signalling: 4414) were diluted 1:1000 in blocking buffer, added to the wells and incubated at 4^o^C on a rocking shelf overnight. ProLong™ Gold Antifade Mountant with DAPI (ThermoFisher Scientific: P36935) was added to each well as mounting media and nuclear staining. The images were processed using ZEN Blue (version 2.3) program from ZEISS.

**Quantification of FANCD2 foci.** Images exported from ZEN Blue were quantitated using ImageJ (<https://imagej.nih.gov/ij/>). The images were converted to 16-bit images, and the background threshold was adjusted and set to to minimize background (0.01-0.02% for the upper limit and the maximum for the lower limit). The “analyze particles” function was used to quantify the number of FAND2 foci in the images using 20 micron per pixel-square as minimum size of a focus. In each image, cells positive for γH2AX foci were first identified visually and then FANCD2 foci was counted in each cell as determined by the ImageJ software. Four images were analysed for each condition, representing an average of 15 cells per image.

1. **SUPPLEMENTARY FIGURES**

**Figure S1. Sanger validation of FA/BRCA-HRR variants.** Results from the Sanger sequencing of gDNA of disease [diagnosis BMMNC (Dx)] and non-disease (mesenchymal stromal cells, MSC) samples are shown as sequencing traces. Cells that are shaded in Blue represent paired (Dx and MSC) traces; cells that are shaded in Green represent MSC only samples; the somatically acquired mutation is shown in Red.

| 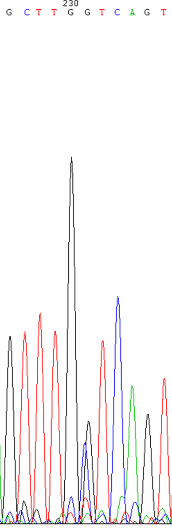DX | 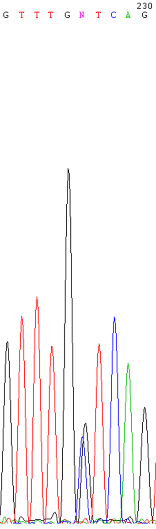MSC | DX 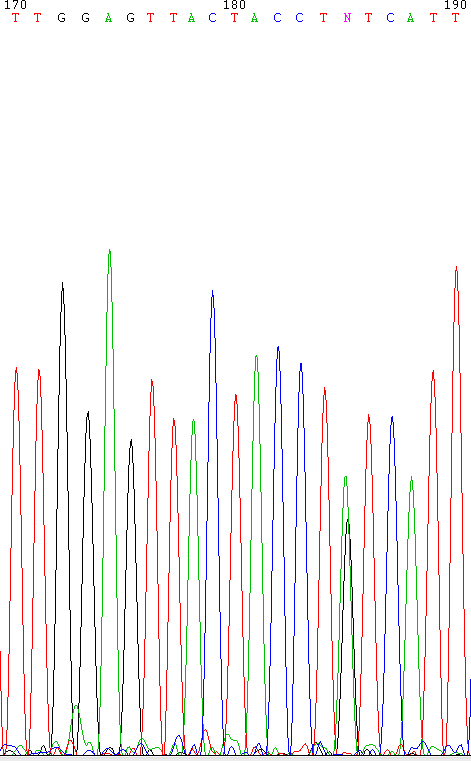 | MSC 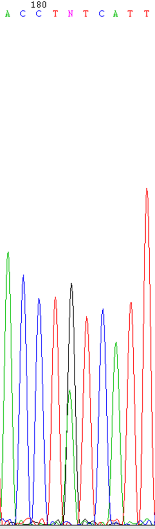 | 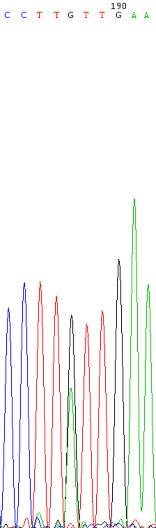DX | MSC 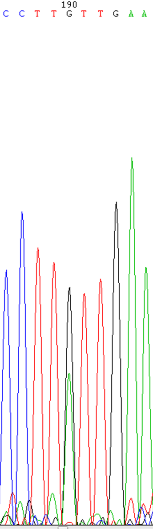 |
| --- | --- | --- | --- | --- | --- |
| FANCA c.C455G | | FANCI c.A394G | | FANCL c.A343G | |
| WES-47 | | WES-59 | | WES-18 | |
| 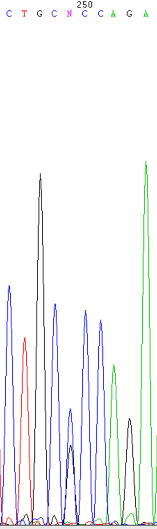DX | MSC 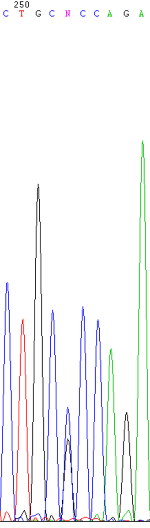 | 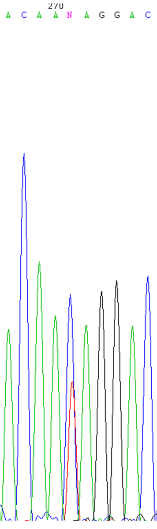DX | 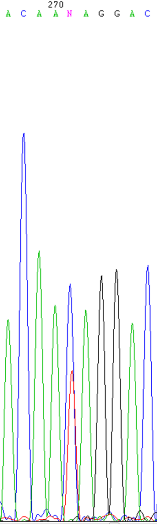MSC | 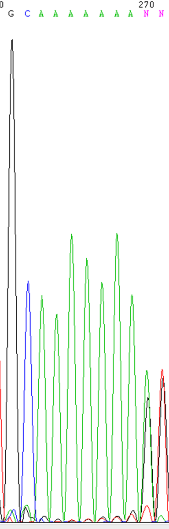DX | 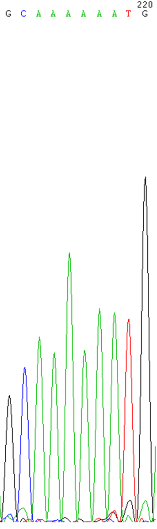MSC |
| FANCL c.C50G | | FANCM c.C5101T | | FANCM c.3998delA | |
| WES-41 | | WES-46 | | WES-94 | |
| 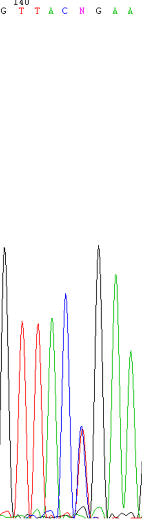DX | 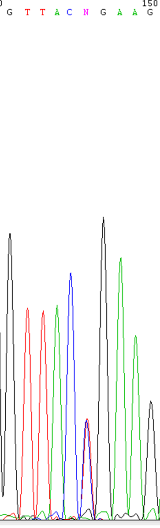MSC | 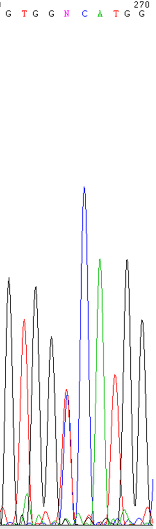DX | 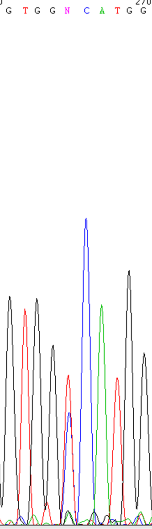MSC |  |  |
| BRCA1 (FANCS) c.C3607T | | SLX4 (FANCP) c.C1271T | |  |  |
| WES-46 | | WES-73 | |  |  |
| 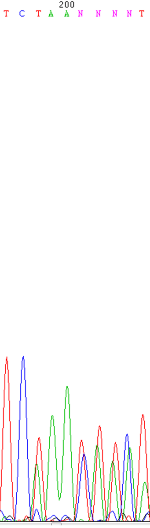MSC | 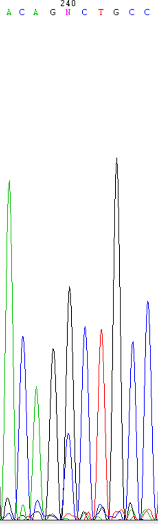MSC | 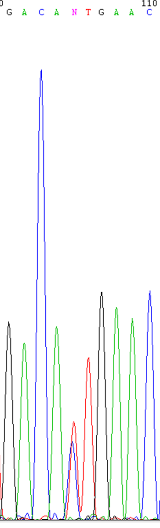MSC |  |  |  |
| BRCA2 (FANCD1) c.6399_6401del | FANCI c.C1656G | ERCC4 (FACNQ) p.I706T |  |  |  |
| WES-32 | WES-1 | WES-34 |  |  |  |

**Figure S2. Expression of mutant FANC variants by Sanger sequencing.** Sanger sequencing traces of cDNA of selected FANC gene variants in AML samples (n=13).

| **Forward sequences** | | **Reverse sequences** | **Forward sequences** | **Reverse sequences** |
| --- | --- | --- | --- | --- |
| 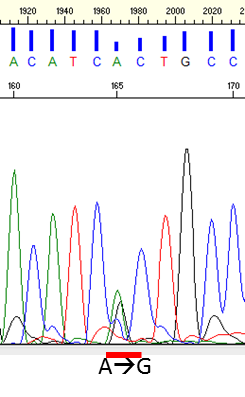 | | 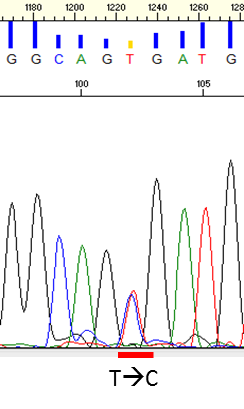 | 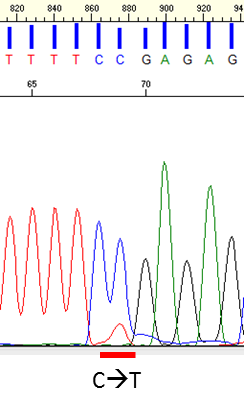 | 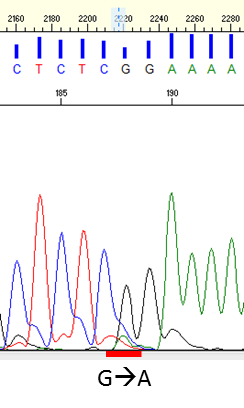 |
| **FANCA - c.A3391G:p.T1131A** | | | **FANCD2 - c.C2776T:p.R926X** | |
| 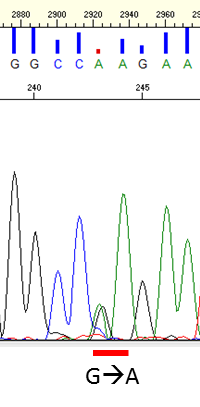 | | 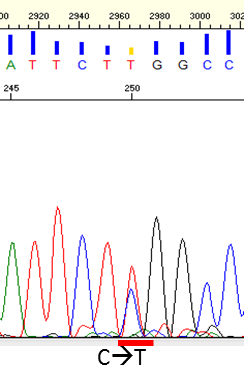 | 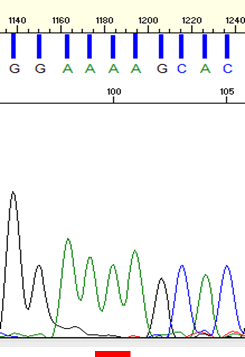 | 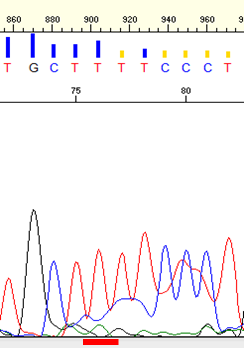 |
| **SLX4 - c.G2437A:p.E813K** | | | **FANCM - c.A1545C:p.K515N** | |
| 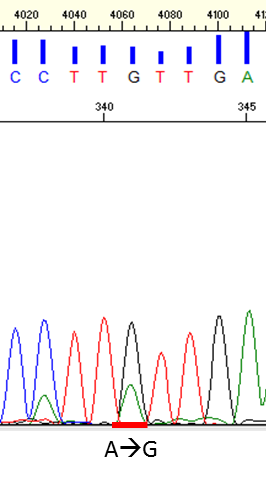 | | 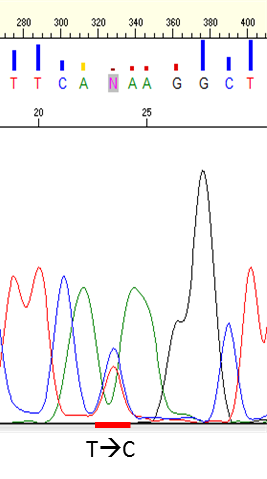 | 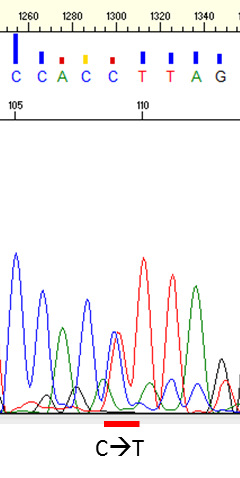 | 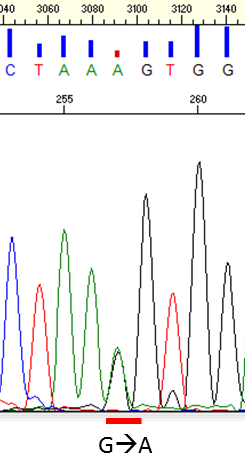 |
| **FANCL - c.A343G:p.I115V** | | | **FANCL - c.C112T:p.L38F** | |
| 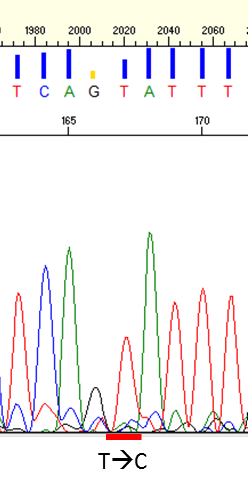 | | 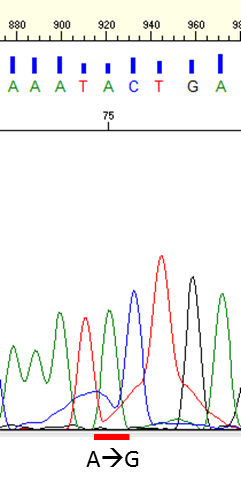 | 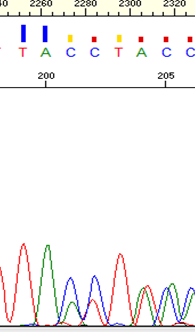 | 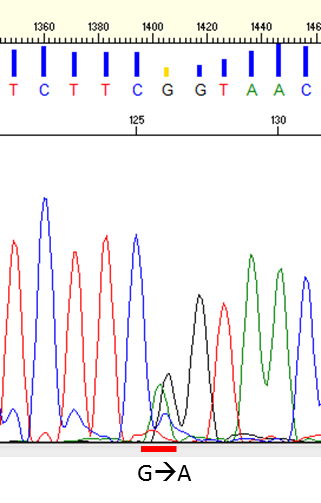 |
| **BRCA1 - c.T2566C:p.Y856H** | | | **FANCL - c.1099_1100insATTA:p.T367fs** | **BRCA1 - c.C3607T:p.R1203X** |
| 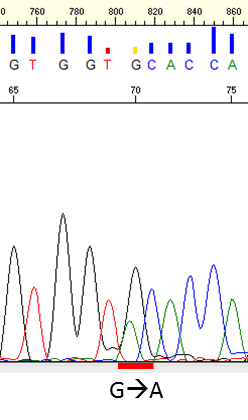 | 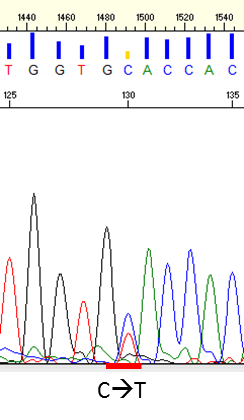 | | 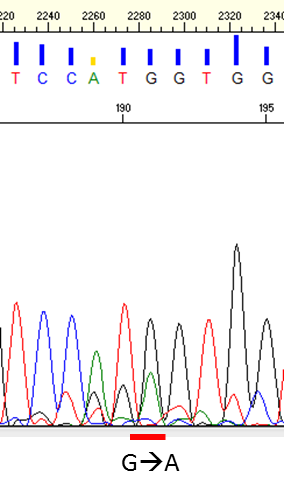 | 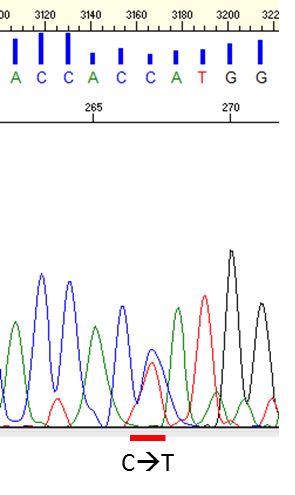 |
| **RAD51C - c.G376A:p.A126T** | | | **BRCA1 - c.G4956A:p.M1652I** | |
| 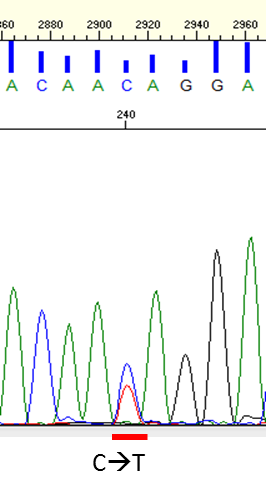 | 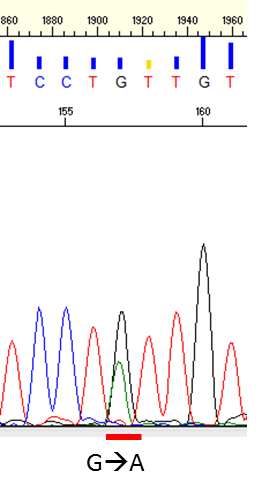 | | 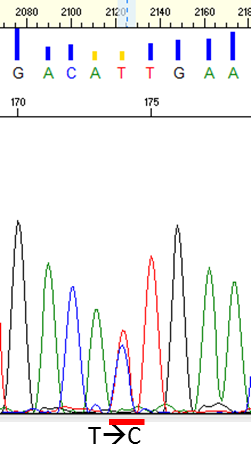 | 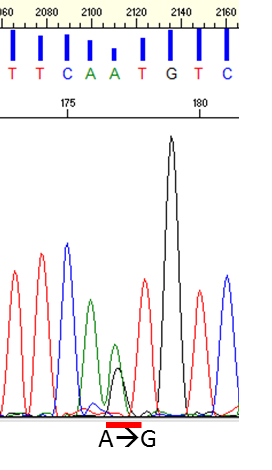 |
| **FANCM - c.C5101T:p.Q1701X** | | | **ERCC4 - c.T2117C:p.I706T** | |

**Figure S3. Mutations identified in *FANCC*, *FANCL* and *FANCM* genes in the Australian AML cohort**. Schematics of protein structures showing mutations in FANCM, FANCL and FANCC identified in diagnostic AML samples. Conserved domains are indicated. Blue circles indicate amino acid substitutions, red circles indicate truncating mutations.

**
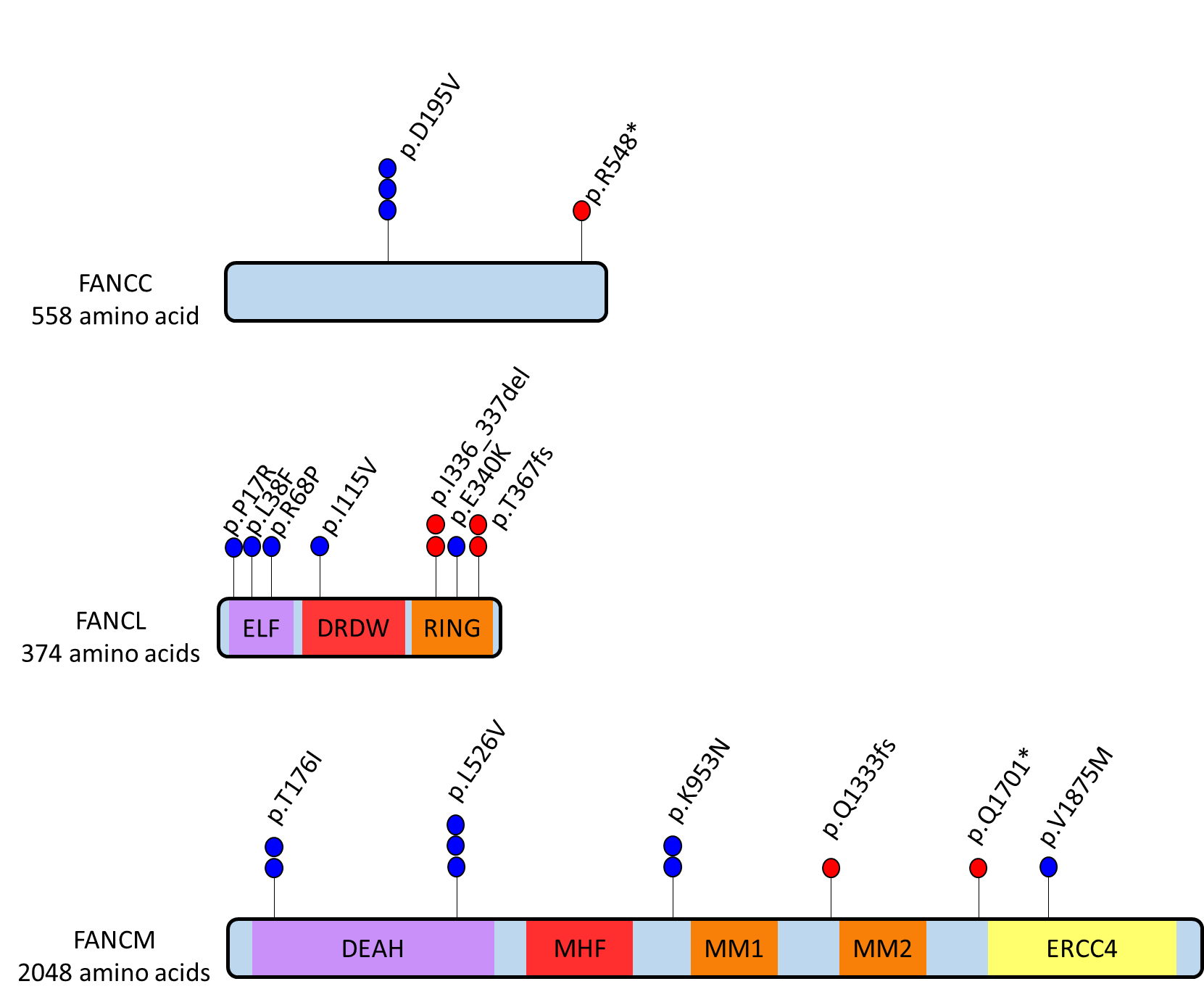
**

**Figure S4. Burden analysis.** Inverse *P* value is plotted for all FANC genes (line represents *P*=0.05).

**Figure S5. MCF10A CRISPR clones.** MCF10A clones generated using CRISPR-Cas9. Blue font represents wild type sequence (identical to the reference sequence) and red font represents the alternate sequence obtained due to frameshift deletions from CRISPR-Cas9. Clone WT is shown to be wild type. Clone Het-1 has a deletion of two nucleotides resulting in a premature stop codon on one allele. Clone Het-2 has a four nucleotide deletion resulting in a premature stop codon on one allele. Clone Het-3 has two consecutive amino acids deleted (glutamine and asparagine) on one allele and was wild type on the second allele. The bi-allelic clone had a premature stop mutation on one allele and an arginine residue deleted on the other allele.

**Figure S6. FANCL expression in MCF10A CRISPR clones.** Western blot for FANCL and tubulin (upper panel), and quantitation bar plot (lower panel), showing reduced FANCL expression in the heterozygous (Het-1, Het-2 and Het-3) and bi-allelic MCF10A CRISPR clones. Tubulin was used as loading control.


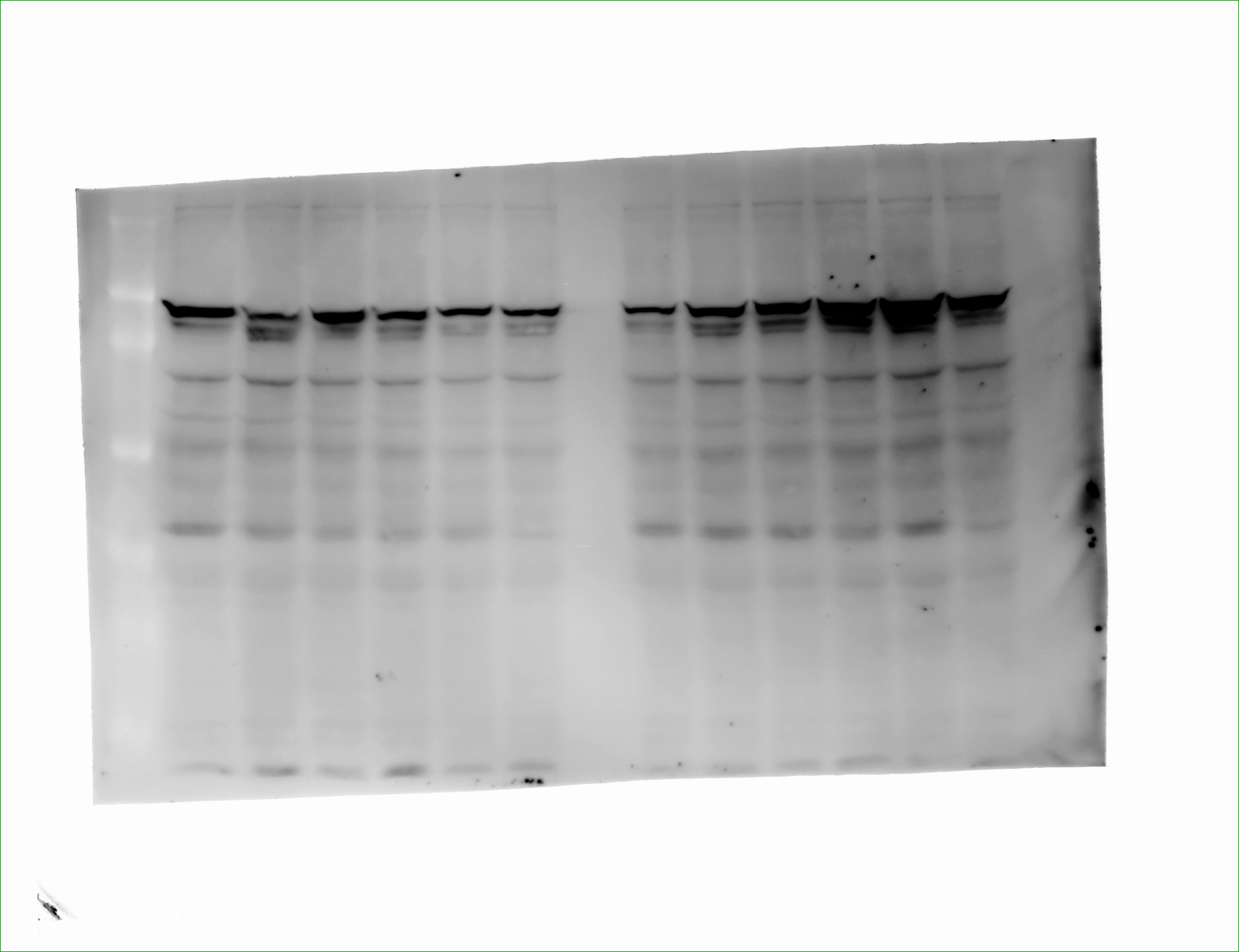

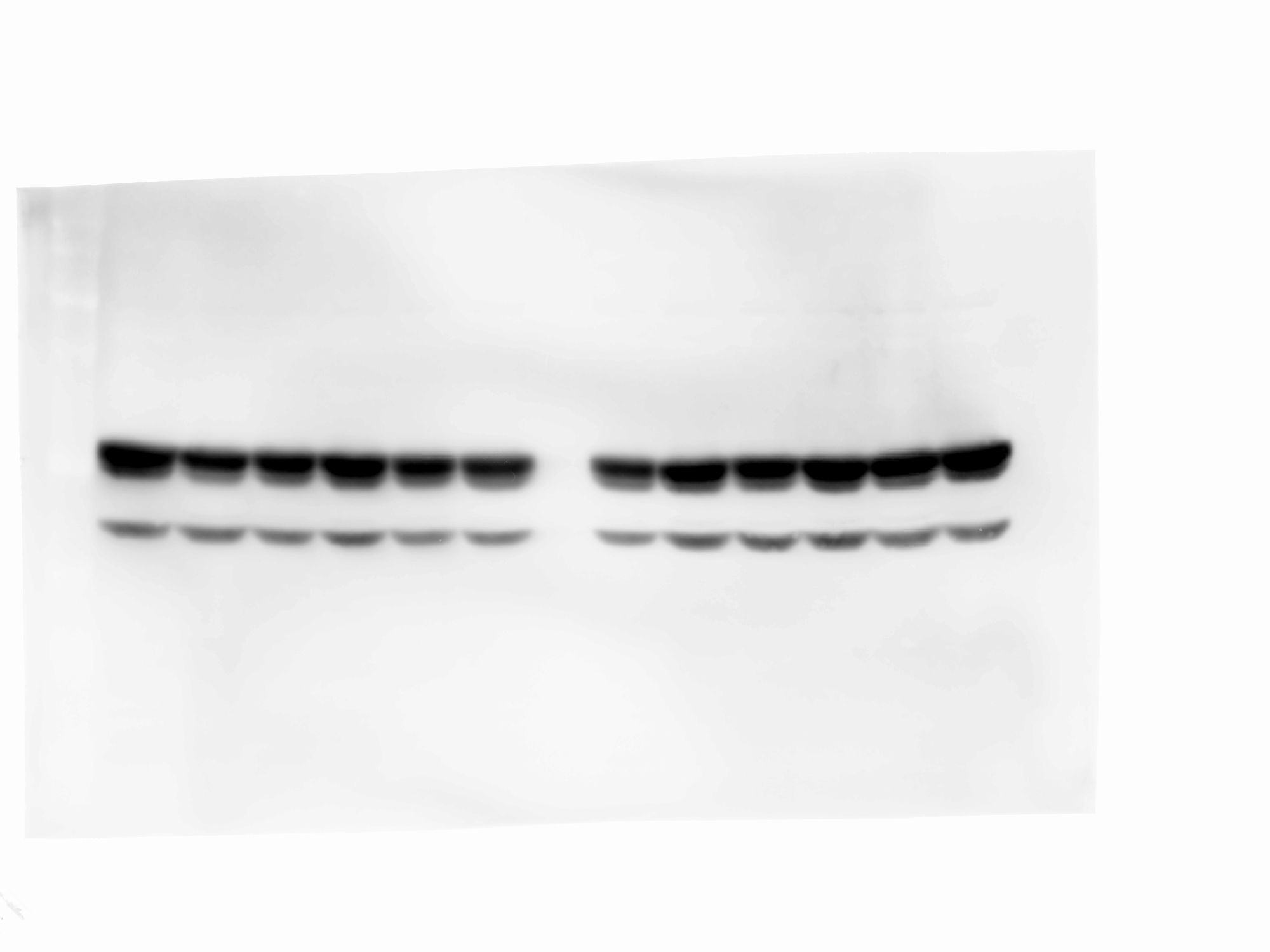


FANCL

Tubulin

WT Het-1 Het-2 Het-3 bi-allelic


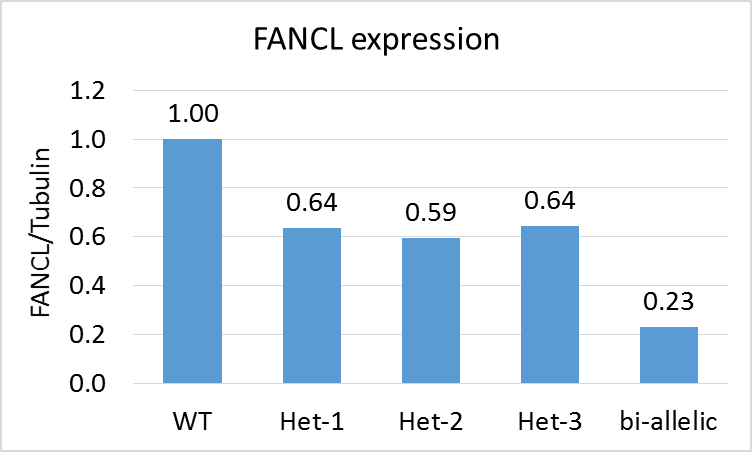


**Figure S7. Cell cycle analysis (n=4).** (**A**) Cell cycle analysis for each of the MCF10A *FANCL* CRISPR clones treated with 40ng/mL of MMC or vehicle (DMSO). Cell cycle analysis showing the G0/G1 phase (**B**), S phase (**C**) and G2/M phase (**D**) for each of the MCF10A *FANCL* CRISPR clones treated with 40ng/mL of MMC or vehicle (DMSO).


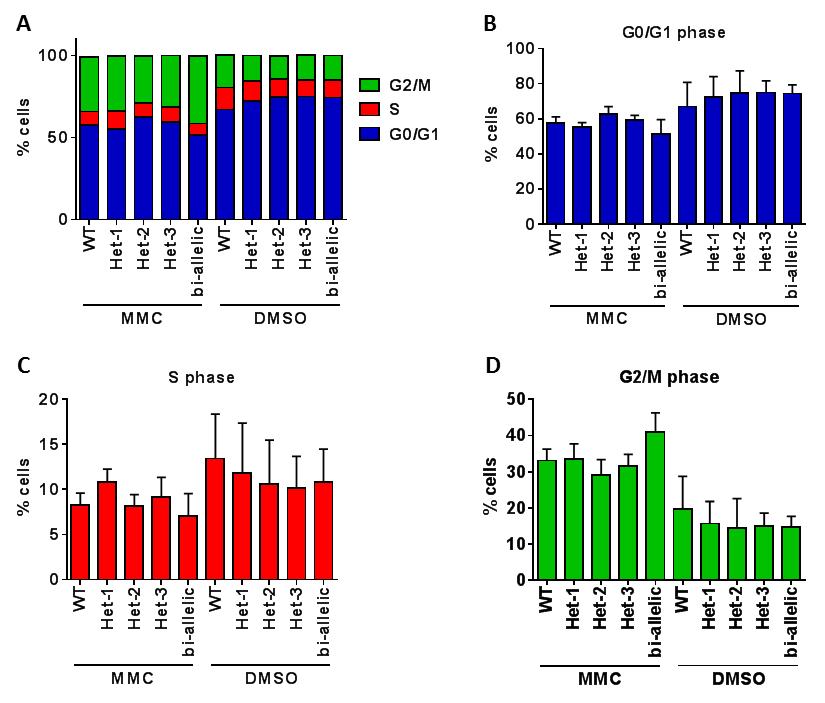


**
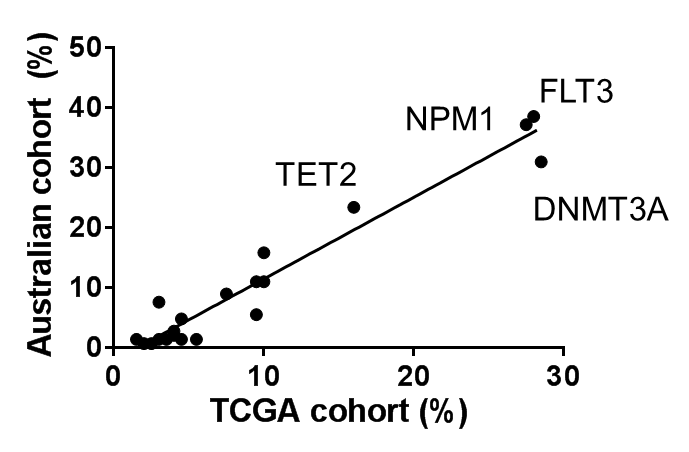
Figure S8. Correlation between percentage of patients in the Australian and TCGA AML cohorts with somatic mutations in genes recurrently mutated in AML (r=0.886).**

**3. SUPPLEMENTARY TABLES**

**Table S1. Characteristics of the Australian AML Cohort**

|  | All Cases  (n=131) | Mutant Group  (n=45) | Non-Mutant Group (n=86) | ^1^P value |
| --- | --- | --- | --- | --- |
| Age - median (range) | 55 (17-89) | 58 (18-84) | 54 (17-89) | 0.8055^ |
| Male – n / total (%) | 80 / 131 (61.1%) | 29 / 45 (64.4%) | 51 / 86 (59.3%) | 0.7062 |
| Female – n / total (%) | 51 / 131 (38.9%) | 16 / 45 (35.6%) | 35 / 86 (40.7%) | 0.7062 |
| WCC x10^9^/L - median (range) | 19 (1.07-313.3) | 26 (1.22-227) | 15.45 (1.07-313.3) | 0.8793^ |
| BM Blast % - median (range) | 80.75 (50-100) | 77 (50-100) | 83.5 (50-99) | 0.2869^ |
| Primary/Secondary AML – n / total (%) |  |  |  |  |
| De Novo | 75 / 83 (90.4%) | 26/30 (86.7%) | 49/53 (92.5%) | 0.4514 |
| Secondary | 8 / 83 (9.6%) | 4 /30(13.3%) | 4/53 (7.5%) | 0.4514 |
| Unknown | 48 |  |  |  |
| Transplant – n / total (%) |  |  |  |  |
| Yes | 23 / 89 (25.8%) | 10 / 32 (31.2%) | 13 / 57 (22.8%) | 0.4521 |
| No | 66 / 89 (74.2%) | 22 / 32 (68.8%) | 44 / 57 (77.2%) | 0.4521 |
| Unknown | 42 |  |  |  |
| ^2^FAB – n / total (%) |  |  |  |  |
| M0 | 4 / 87 (4.6%) | 0 / 32 (0%) | 4 / 55 (7.3%) | 0.2932 |
| M1 | 31 / 87 (35.6%) | 9 / 32 (28.1%) | 22 / 55 (40%) | 1 |
| M2 | 17 / 87 (19.5%) | 7 / 32 (21.9%) | 10 / 55 (18.2%) | 0.7808 |
| M3 | 0 / 87 (0%) | 0 / 32 (0%) | 0 / 55 (0%) | 1 |
| M4 | 17 / 87 (19.5%) | 8 / 32 (25%) | 9 / 55 (16.4%) | 0.4032 |
| M5 | 13 / 87 (14.9%) | 5 / 32 (15.6%) | 8 / 55 (14.5%) | 1 |
| M6 | 0 / 87 (0%) | 0 / 32 (0%) | 0 / 55 (0%) | 1 |
| M7 | 1 / 87 (1.1%) | 1 / 32 (3.1%) | 0 / 55 (0%) | 0.3678 |
| Not classified | 4 / 87 (4.6%) | 2 / 32 (6.3%) | 2 / 55 (3.6%) | 0.6196 |
| Unknown | 44 |  |  |  |
| ^3^ELN Cytogenetic Risk – n/total (%) |  |  |  |  |
| Good | 11 / 54 (20.4%) | 5 / 21 (23.8%) | 6 / 33 (18.2%) | 0.7329 |
| Intermediate-1 | 11 / 54 (20.4%) | 3 / 21 (14.3 %) | 8 / 33 (24.2%) | 0.4974 |
| Intermediate-2 | 13 / 54 (24.1%) | 9 / 21 (42.9%) | 4 / 33 (12.1%) | **0.02*** |
| Adverse | 19 / 54 (35.2%) | 4 / 21 (19%) | 15 / 33 (45.5%) | 0.0786 |
| Unknown | 77 |  |  |  |
| ^4^Grimwade Cytogenetic Risk – n/total (%) |  |  |  |  |
| Good | 7 / 122 (5.7%) | 2 / 40 (5%) | 5 / 82 (6.1%) | 1 |
| Intermediate | 88 / 122 (72.1%) | 31 / 40 (77.5%) | 57 / 82 (69.5%) | 0.3970 |
| Poor | 27 / 122 (22.1%) | 7 /40 (17.5%) | 20 / 82 (24.4%) | 0.4886 |
| Unknown | 9 |  |  |  |
| Simple Karyotype – n/total (%) |  |  |  |  |
| Normal | 70 / 129 (54.3%) | 20 / 44 (44.5%) | 50 / 85 (58.8%) | 0.1921 |
| Abnormal | 40 / 129 (31.0%) | 18 / 44 (40.9%) | 22 / 85 (25.9%) | 0.1079 |
| Complex | 19 / 129 (14.7%) | 6 / 44 (13.6%) | 13 / 85 (15.3%) | 1 |
| Unknown | 2 |  |  |  |
| Cytogenetics – n/total (%) |  |  |  |  |
| t(15;17) | 0 / 129 (0%) | 0 (0%) | 0 (0%) | 1 |
| CBF | 10 / 129 (7.75%) | 3 / 44 (6.82%) | 7 / 85 (8.24%) | 1 |
| MLL | 7 / 129 (5.43%) | 3 / 44 (6.82%) | 4 / 85 (4.71%) | 0.6895 |
| tri(8) | 11 / 129 (8.53%) | 3 / 44 (6.82%) | 8 / 85 (9.41%) | 0.7481 |
| mono(5) / del(5q) | 5 / 129 (3.88%) | 1 / 44 (2.27%) | 4 / 85 (4.71%) | 0.6605 |
| mono(7) / del(7q) | 9 / 129 (6.98%) | 0 / 44 (0%) | 9 / 85 (10.59%) | **0.0275*** |
| tri(21) | 3 / 129 (2.33%) | 1 / 44 (2.27%) | 2 / 85 (2.35%) | 1 |
| Mutations – n/total (%) |  |  |  |  |
| FLT3-ITD | 44 / 131 (33.6%) | 10 / 45 (22.2%) | 34 / 86 (39.5%) | 0.0531 |
| FLT3-TKD | 7 / 131 (5.34%) | 4 / 45 (8.9%) | 3 / 86 (3.5%) | 0.2315 |
| NPM1 | 46 / 131 (35.1%) | 12 / 45 (26.7%) | 34 / 86 (39.5%) | 0.1784 |
| DNMT3A | 41 / 131 (31.3%) | 15 / 45 (33.3%) | 26 / 86 (30.2%) | 0.8429 |
| IDH1 | 14 / 131 (10.7%) | 6 / 45 (13.3%) | 8 / 86 (9.3%) | 0.5549 |
| IDH2 | 24 / 131 (18.3%) | 9 / 45 (20%) | 15 / 86 (17.4%) | 0.8128 |
| TET2 | 21 / 131 (16%) | 7 / 45 (15.6%) | 14 / 86 (16.3%) | 1 |

^1^P values are calculated by Fisher’s exact test except for: ^ determined by Student’s *t*-test.

^2^FAB: French-America-British classification ^9^

^3^ELN: European LeukaemiaNet ^10^

^4^Grimwade classification ^11^

**P*<0.05

**Table S2. List of FANC variants in AML (Australian cohort)**

Refer to “Supplementary Table S2.xlsx”

**Table S3. Selected FA Core and ID2 gene variants of interest in the Australian AML cohort**

| Gene | AML Mutation | Comments |
| --- | --- | --- |
| *FANCA* | p.T1131A | Reported in MDS/sAML.^12^ |
|  | p.R1144W | Known FA compound heterozygous mutation.^13^ |
| *FANCC* | p.R548X | Occurs in multiple FA patients; severe phenotype;^14^ detected in both Australian and TCGA cohorts. |
|  | p.D195V | Reported mutation in FA.^15^ |
| *FANCD2* | p.R926X | Known FA compound heterozygous variant.^16^ Recurrent somatic truncations at this residue in solid tumours (COSMIC). |
|  | c.2715+1G>A | Known FA compound heterozygous variant.^16^ |
| *FANCL* | p.336_337del | Known RING domain null mutation.^17^ |
|  | p.T367fs | Frame shift occurs at the same position as reported in the FA database (ID: FANCL_000003). Present in two patients in the AML cohort with early onset of disease (27 and 46 years old). |
|  | p.E340K | Ring domain charge-reversal, affects the binding of *FANCL* to *FANCT.*^18^ |
|  | p.L38F | Patient presented with anemia and neutropenia prior to AML diagnosis. |
|  | p.I115V | Patient presented with neutropenia and MDS prior to AML diagnosis. |
|  | p.P17R | Patient presented with mild neutropenia prior to AML diagnosis. |
| *FANCM* | p.Q1701X | Enriched in patients with triple-negative breast cancer.^19^ Also identified in a paediatric B-ALL case.^20^ |
|  | p.V1857M | Reported in a breast cancer family.^21^ |

**Table S4. Frequency of FANC variants in ExAC and Australian AML cohorts.**

|  | **^1^ExAC (n=66740)** | | | **^1^AML (n=262)** | | |  |
| --- | --- | --- | --- | --- | --- | --- | --- |
| **Gene** | **Count** | **ExAC Frequency** | **Frequency (%)** | **Count** | **AML Frequency** | **Frequency (%)** | **^2^P-value** |
| FANCA | 653 | 0.0098 | 0.9784 | 2 | 0.0076 | 0.7634 | 0.9693 |
| FANCB | 145 | 0.0022 | 0.2173 | 0 | 0 | 0 | 0.9289 |
| **FANCC** | **211** | **0.0032** | **0.3162** | **4** | **0.0153** | **1.5267** | **0.0036*** |
| BRCA2 | 1276 | 0.0191 | 1.9119 | 3 | 0.0115 | 1.145 | 0.497 |
| FANCD2 | 546 | 0.0082 | 0.8181 | 2 | 0.0076 | 0.7634 | 0.8061 |
| FANCE | 302 | 0.0045 | 0.4525 | 1 | 0.0038 | 0.3817 | 0.7712 |
| FANCF | 178 | 0.0027 | 0.2667 | 0 | 0 | 0 | 0.8136 |
| FANCG | 219 | 0.0033 | 0.3281 | 1 | 0.0038 | 0.3817 | 0.6967 |
| FANCI | 762 | 0.0114 | 1.1417 | 3 | 0.0115 | 1.145 | 0.7746 |
| BRIP1 | 406 | 0.0061 | 0.6083 | 1 | 0.0038 | 0.3817 | 0.9419 |
| **FANCL** | **321** | **0.0048** | **0.481** | **9** | **0.0344** | **3.4351** | **< 0.0001*** |
| **FANCM** | **792** | **0.0119** | **1.1867** | **10** | **0.0382** | **3.8168** | **0.0003*** |
| PALB2 | 322 | 0.0048 | 0.4825 | 2 | 0.0076 | 0.7634 | 0.8353 |
| RAD51C | 157 | 0.0024 | 0.2352 | 1 | 0.0038 | 0.3817 | 0.8805 |
| SLX4 | 689 | 0.0103 | 1.0324 | 6 | 0.0229 | 2.2901 | 0.0892 |
| ERCC4 | 669 | 0.01 | 1.0024 | 4 | 0.0153 | 1.5267 | 0.5899 |
| RAD51 | 94 | 0.0014 | 0.1408 | 0 | 0 | 0 | 0.8266 |
| BRCA1 | 619 | 0.0093 | 0.9275 | 3 | 0.0115 | 1.145 | 0.9651 |
| UBE2T | 108 | 0.0016 | 0.1618 | 0 | 0 | 0 | 0.9046 |
| XRCC2 | 97 | 0.0015 | 0.1453 | 0 | 0 | 0 | 1 |
| MAD2L2 | 59 | 0.0009 | 0.0884 | 0 | 0 | 0 | 1 |
| RFWD3 | 281 | 0.0042 | 0.421 | 1 | 0.0038 | 0.3817 | 1 |

^1^ n represents the total number of alleles in each cohort. The non-Finnish European cohort in ExAC consists of 33370 individuals.

^2^ Fisher’s exact test was used to determine the statistical difference between the Australian AML cohort and the non-Finnish European cohort in ExAC. (* P<0.05).

**Table S5. FANC variants as risk alleles in cancer studies.**

| **Type of Cancer** | **Gene** | **Observation** | **OR** |
| --- | --- | --- | --- |
| Triple negative breast cancer^19,22^ | *FANCM*^19^ | Increased incidence of triple negative breast cancer. | 3.56 |
|  | *FANCL*^22^ |  | - |
| Metastatic castrate-resistant prostate cancer^23^ | *FANCL* | Pathogenic and germline mutations. | - |
|  | *BRCA2* |  | - |
| Breast cancer^24^ | *FANCC* | Predisposing gene for breast cancer across 2 families (4 generations). Mutational hotspot identified. | - |
| Therapy related myeloid neoplasms^25^ | *FANCA* | Primary tumor: breast and non-Hodgkin lymphoma. | - |
|  | *FANCD2* | Primary tumor: Hodgkin lymphoma. | - |
|  | *FANCJ, FANCC* | Same patient carries mutation in both genes in breast and adrenal primary tumors. | - |
| sAML^12^ | *BRCA2* | Heterozygous pathogenic germline mutations. | - |
|  | *FANCI* |  | - |
| MDS^12^ | *FANCA* | Heterozygous pathogenic germline mutations. | 4.9 |
|  | *FANCG* |  | 5.9 |
|  | *BRCA2* |  | - |
|  | *FANCC* |  | - |
|  | *FANCE* |  | - |
|  | *FANCI* |  | - |
|  | *FANCL* |  | - |
|  | *RAD51C* |  | - |
| Colorectal cancer^26^ | *FANCC* | Pathogenic variants segregates with disease in the 6 families. | - |
|  | *FANCE* |  | - |
|  | *BRIP1* |  | - |
|  | *BRCA2* |  | - |
| Pediatric cancer^20^ | *BRCA2* | Pathogenic and germline mutations. | - |
| Pan cancers^27^ | *BRCA2* | Enrichment of truncation variants was observed in comparison to normal healthy controls. | - |
|  | *BRCA1* |  | - |
|  | *PALB2* |  | - |
|  | *RAD51C* |  | - |

**4. SUPPLEMENTARY REFERENCES**

1. Duncan EL, Danoy P, Kemp JP, Leo PJ, McCloskey E, Nicholson GC*, et al.* Genome-wide association study using extreme truncate selection identifies novel genes affecting bone mineral density and fracture risk. *PLoS Genet* 2011; **7**: e1001372.

2. Li H, Homer N. A survey of sequence alignment algorithms for next-generation sequencing. *Brief Bioinform* 2010; **11**: 473-483.

3. Li H, Handsaker B, Wysoker A, Fennell T, Ruan J, Homer N*, et al.* The Sequence Alignment/Map format and SAMtools. *Bioinformatics* 2009; **25**: 2078-2079.

4. Liu X, Jian X, Boerwinkle E. dbNSFP: a lightweight database of human nonsynonymous SNPs and their functional predictions. *Hum Mutat* 2011; **32**: 894-899.

5. Kircher M, Witten DM, Jain P, O'Roak BJ, Cooper GM, Shendure J. A general framework for estimating the relative pathogenicity of human genetic variants. *Nat Genet* 2014; **46**: 310-315.

6. Cancer Genome Atlas Research N. Genomic and epigenomic landscapes of adult de novo acute myeloid leukemia. *N Engl J Med* 2013; **368**: 2059-2074.

7. Lee S, Emond MJ, Bamshad MJ, Barnes KC, Rieder MJ, Nickerson DA*, et al.* Optimal unified approach for rare-variant association testing with application to small-sample case-control whole-exome sequencing studies. *Am J Hum Genet* 2012; **91**: 224-237.

8. Moslehi R, Singh R, Lessner L, Friedman JM. Impact of BRCA mutations on female fertility and offspring sex ratio. *Am J Hum Biol* 2010; **22**: 201-205.

9. Neame PB, Soamboonsrup P, Browman GP, Meyer RM, Benger A, Wilson WE*, et al.* Classifying acute leukemia by immunophenotyping: a combined FAB-immunologic classification of AML. *Blood* 1986; **68**: 1355-1362.

10. Dohner H, Estey EH, Amadori S, Appelbaum FR, Buchner T, Burnett AK*, et al.* Diagnosis and management of acute myeloid leukemia in adults: recommendations from an international expert panel, on behalf of the European LeukemiaNet. *Blood* 2010; **115**: 453-474.

11. Grimwade D, Hills RK, Moorman AV, Walker H, Chatters S, Goldstone AH*, et al.* Refinement of cytogenetic classification in acute myeloid leukemia: determination of prognostic significance of rare recurring chromosomal abnormalities among 5876 younger adult patients treated in the United Kingdom Medical Research Council trials. *Blood* 2010; **116**: 354-365.

12. Przychodzen B, Makishima H, Sekeres MA, Balasubramanian SK, Thota S, Patel BJ*, et al.* Fanconi Anemia germline variants as susceptibility factors in aplastic anemia, MDS and AML. *Oncotarget* 2018; **9**: 2050-2057.

13. Gille JJ, Floor K, Kerkhoven L, Ameziane N, Joenje H, de Winter JP. Diagnosis of Fanconi Anemia: Mutation Analysis by Multiplex Ligation-Dependent Probe Amplification and PCR-Based Sanger Sequencing. *Anemia* 2012; **2012**: 603253.

14. Lo ten Foe JR, Barel MT, Thuss P, Digweed M, Arwert F, Joenje H. Sequence variations in the Fanconi anaemia gene, FAC: pathogenicity of 1806insA and R548X and recognition of D195V as a polymorphic variant. *Hum Genet* 1996; **98**: 522-523.

15. Verlander PC, Lin JD, Udono MU, Zhang Q, Gibson RA, Mathew CG*, et al.* Mutation analysis of the Fanconi anemia gene FACC. *Am J Hum Genet* 1994; **54**: 595-601.

16. Kalb R, Neveling K, Hoehn H, Schneider H, Linka Y, Batish SD*, et al.* Hypomorphic mutations in the gene encoding a key Fanconi anemia protein, FANCD2, sustain a significant group of FA-D2 patients with severe phenotype. *Am J Hum Genet* 2007; **80**: 895-910.

17. Ali AM, Kirby M, Jansen M, Lach FP, Schulte J, Singh TR*, et al.* Identification and characterization of mutations in FANCL gene: a second case of Fanconi anemia belonging to FA-L complementation group. *Hum Mutat* 2009; **30**: E761-770.

18. Hodson C, Purkiss A, Miles JA, Walden H. Structure of the human FANCL RING-Ube2T complex reveals determinants of cognate E3-E2 selection. *Structure* 2014; **22**: 337-344.

19. Kiiski JI, Pelttari LM, Khan S, Freysteinsdottir ES, Reynisdottir I, Hart SN*, et al.* Exome sequencing identifies FANCM as a susceptibility gene for triple-negative breast cancer. *Proc Natl Acad Sci U S A* 2014; **111**: 15172-15177.

20. Zhang J, Walsh MF, Wu G, Edmonson MN, Gruber TA, Easton J*, et al.* Germline Mutations in Predisposition Genes in Pediatric Cancer. *N Engl J Med* 2015; **373**: 2336-2346.

21. Garcia MJ, Fernandez V, Osorio A, Barroso A, Fernandez F, Urioste M*, et al.* Mutational analysis of FANCL, FANCM and the recently identified FANCI suggests that among the 13 known Fanconi Anemia genes, only FANCD1/BRCA2 plays a major role in high-risk breast cancer predisposition. *Carcinogenesis* 2009; **30**: 1898-1902.

22. Lhota F, Zemankova P, Kleiblova P, Soukupova J, Vocka M, Stranecky V*, et al.* Hereditary truncating mutations of DNA repair and other genes in BRCA1/BRCA2/PALB2-negatively tested breast cancer patients. *Clin Genet* 2016; **90**: 324-333.

23. Hart SN, Ellingson MS, Schahl K, Vedell PT, Carlson RE, Sinnwell JP*, et al.* Determining the frequency of pathogenic germline variants from exome sequencing in patients with castrate-resistant prostate cancer. *BMJ Open* 2016; **6**: e010332.

24. Thompson ER, Doyle MA, Ryland GL, Rowley SM, Choong DY, Tothill RW*, et al.* Exome sequencing identifies rare deleterious mutations in DNA repair genes FANCC and BLM as potential breast cancer susceptibility alleles. *PLoS Genet* 2012; **8**: e1002894.

25. Voso MT, Fabiani E, Zang Z, Fianchi L, Falconi G, Padella A*, et al.* Fanconi anemia gene variants in therapy-related myeloid neoplasms. *Blood Cancer J* 2015; **5**: e323.

26. Esteban-Jurado C, Franch-Exposito S, Munoz J, Ocana T, Carballal S, Lopez-Ceron M*, et al.* The Fanconi anemia DNA damage repair pathway in the spotlight for germline predisposition to colorectal cancer. *Eur J Hum Genet* 2016; **24**: 1501-1505.

27. Lu C, Xie M, Wendl MC, Wang J, McLellan MD, Leiserson MD*, et al.* Patterns and functional implications of rare germline variants across 12 cancer types. *Nat Commun* 2015; **6**: 10086.
